# Masked Mastoiditis Presenting As Pneumococcal Meningoencephalitis in an Elderly Patient: A Diagnostic Pitfall in the Emergency Department

**DOI:** 10.7759/cureus.105079

**Published:** 2026-03-11

**Authors:** Carlos M Bandera-Flores, Begoña Zalba-Etayo, Violeta Bergua-Díez, María del Carmen Lahoza-Pérez, Daniel Sáenz-Abad

**Affiliations:** 1 Department of Medicine, Universidad Autónoma del Estado de Morelos, Cuernavaca, MEX; 2 Department of Medicine, Universidad de Zaragoza, Zaragoza, ESP; 3 Emergency Department, Hospital Clinico Universitario Lozano Blesa, Zaragoza, ESP; 4 Cardiovascular Medicine, Instituto de Investigación Sanitaria Aragón, Zaragoza, ESP; 5 Emergency Department, Hospital Clínico Universitario Lozano Blesa, Zaragoza, ESP

**Keywords:** diagnostic pitfall, elderly patient, emergency department, intracranial abscess, masked mastoiditis, otogenic infection, pneumococcal meningoencephalitis

## Abstract

Otogenic infections remain a potentially under-recognized cause of pneumococcal meningoencephalitis in adults. In elderly patients, acute mastoiditis may present without overt otological findings (“masked mastoiditis”), delaying diagnosis and increasing the risk of intracranial complications. An 86-year-old unvaccinated male patient with multiple comorbidities initially presented to the emergency department with non-specific upper respiratory symptoms and normal otoscopy and was discharged with presumed viral infection. Four days later, he returned with acute neurological deterioration and hemodynamic instability. Cranial computed tomography demonstrated left mastoid and middle ear opacification with petrous bone erosion and suspected intracranial extension. Magnetic resonance imaging confirmed a left temporobasal abscess. Blood cultures and urinary antigen testing were positive for Streptococcus pneumoniae. The patient was diagnosed with pneumococcal meningoencephalitis secondary to acute mastoiditis and was treated with intravenous antibiotics, corticosteroids, intensive care support, and surgical mastoid drainage. The hospital course was prolonged, with persistent cognitive impairment at discharge. Masked mastoiditis may lead to life-threatening intracranial infection despite normal otoscopic findings. In elderly patients presenting with fever and altered mental status, clinicians should consider occult otogenic sources. Early neuroimaging is crucial when lumbar puncture is contraindicated or delayed, particularly in clinically unstable patients.

## Introduction

Acute bacterial meningitis remains a life-threatening condition, particularly in elderly patients and those with chronic comorbidities. Streptococcus pneumoniae continues to be a leading cause of invasive bacterial infection in adults, including meningitis [1].

Otogenic infections represent an important and potentially under-recognized source of pneumococcal meningitis in adults. Although acute mastoiditis predominantly affects children, adult cases are associated with a higher risk of intracranial complications, including meningitis, subdural empyema, and brain abscess [2]. In some patients, mastoid infection may occur without overt otological findings, a presentation referred to as masked mastoiditis. This atypical form can delay recognition of the infectious focus, particularly in elderly individuals presenting with non-specific symptoms. In such cases, normal otoscopic findings may provide false reassurance and postpone appropriate imaging and treatment [2-5].

Neuroimaging plays a central role in the diagnosis of otogenic intracranial infection, especially when clinical findings are non-specific or misleading [6,7].

We report the case of an elderly, unvaccinated patient who developed severe pneumococcal meningoencephalitis secondary to acute mastoiditis, initially presenting with non-specific symptoms and normal otoscopic findings. Although mastoiditis is an uncommon cause of meningitis in adults, masked mastoiditis may delay recognition of the infectious focus and lead to severe intracranial complications. This case highlights the diagnostic challenges posed by this condition and underscores the importance of maintaining clinical suspicion and performing early neuroimaging when symptoms remain unexplained.

## Case presentation

An 86-year-old male patient with a history of type 2 diabetes mellitus, hypertension, and dyslipidaemia, previously independent in activities of daily living, presented to the emergency department (ED) with fever, cough, and pharyngeal pain. Otoscopic examination performed in the ED revealed no signs of otitis media or externa, and there was no otalgia or otorrhoea. He was discharged with symptomatic treatment (acetaminophen) and a diagnosis of presumed viral upper respiratory tract infection.

Four days later, the patient re-presented to the ED with an acute decreased level of consciousness. On examination, he was febrile (38.8 °C; reference values: 36.5-37.2°C) and hemodynamically unstable, with hypotension (blood pressure 88/62 mmHg; reference values: 90-120/60-80 mmHg), tachycardia (heart rate 138 beats per minute; reference values: 60-100 beats per minute), and tachypnea (respiratory rate 26 breaths per minute; reference values: 12-20 breaths per minute). Neurological examination revealed a markedly reduced level of consciousness without focal neurological deficits. Otoscopic examination again showed no abnormalities, and no retroauricular swelling or tenderness was observed. Despite the absence of overt otological findings, the severity of the neurological presentation and the lack of an alternative infectious focus raised suspicion of a possible occult otogenic source.

Laboratory investigations demonstrated leukocytosis, markedly elevated inflammatory markers, hyperglycemia, and progressive renal dysfunction (Table 1).

**Table 1:** Laboratory investigations during hospitalization WBCC: White Blood Cell Count

Parameter	Admission	24 hours	48 hours	Reference range
Hemoglobin (g/dL)	14.2	11.3	11.7	13-17.4
WBCC (/mm^3^)	14,500	12,700	7,900	4,000-11,000
Neutrophils (/mm^3^)	13,400	11,900	7,300	1,600-7,000
Glucose (mg/dL)	344	354	203	82-115
Blood urea nitrogen (mg/dL)	65	95	114	18-55
Creatinine (mg/dL)	1.67	1.74	2.02	0.7-1.2
Sodium (mmol/L)	135	141	143	136-145
Potassium (mmol/L)	4.99	4.66	4.55	3.5-5.1
Fibrinogen (mg/dL)	921	907	827	200-400
C-reactive protein (mg/L)	228	>350	239	0.1-5
Procalcitonin (µg/L)	1.95	13.6	9.7	<0.5
Lactate (mmol/L)	2.7	2.4	2.1	0.5-2.2

**Figure 1:**
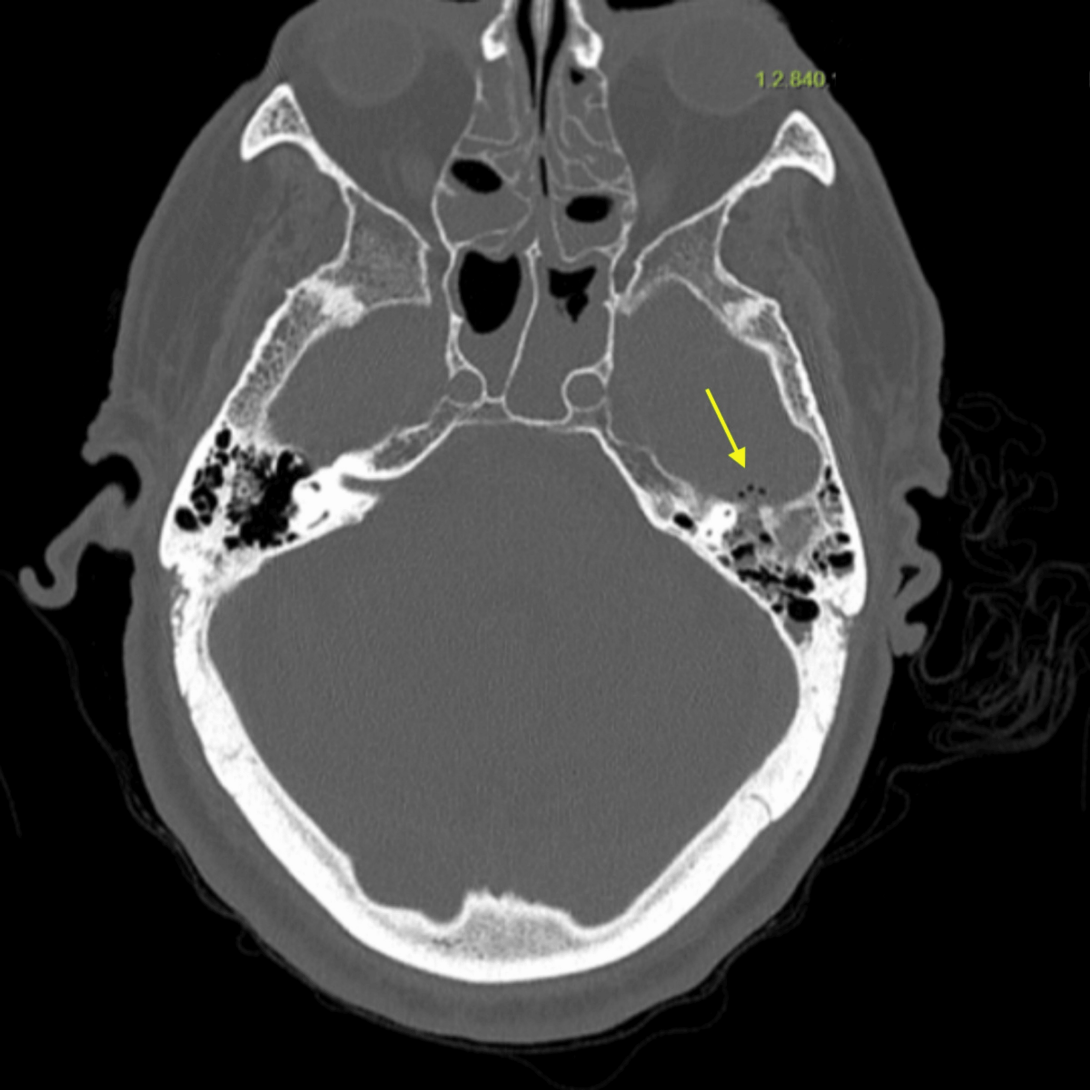
Cranial computed tomography (bone window) Bone-window images showing opacification of the left mastoid air cells and middle ear cavity, with cortical disruption of the anterior wall of the left petrous bone (yellow arrow).

Cranial computed tomography (CT) was therefore requested to evaluate for potential intracranial pathology and to rule out masked mastoiditis. Imaging demonstrated opacification of the left mastoid air cells and middle ear cavity, with cortical disruption of the anterior wall of the left petrous bone (Figure 1).

**Figure 2:**
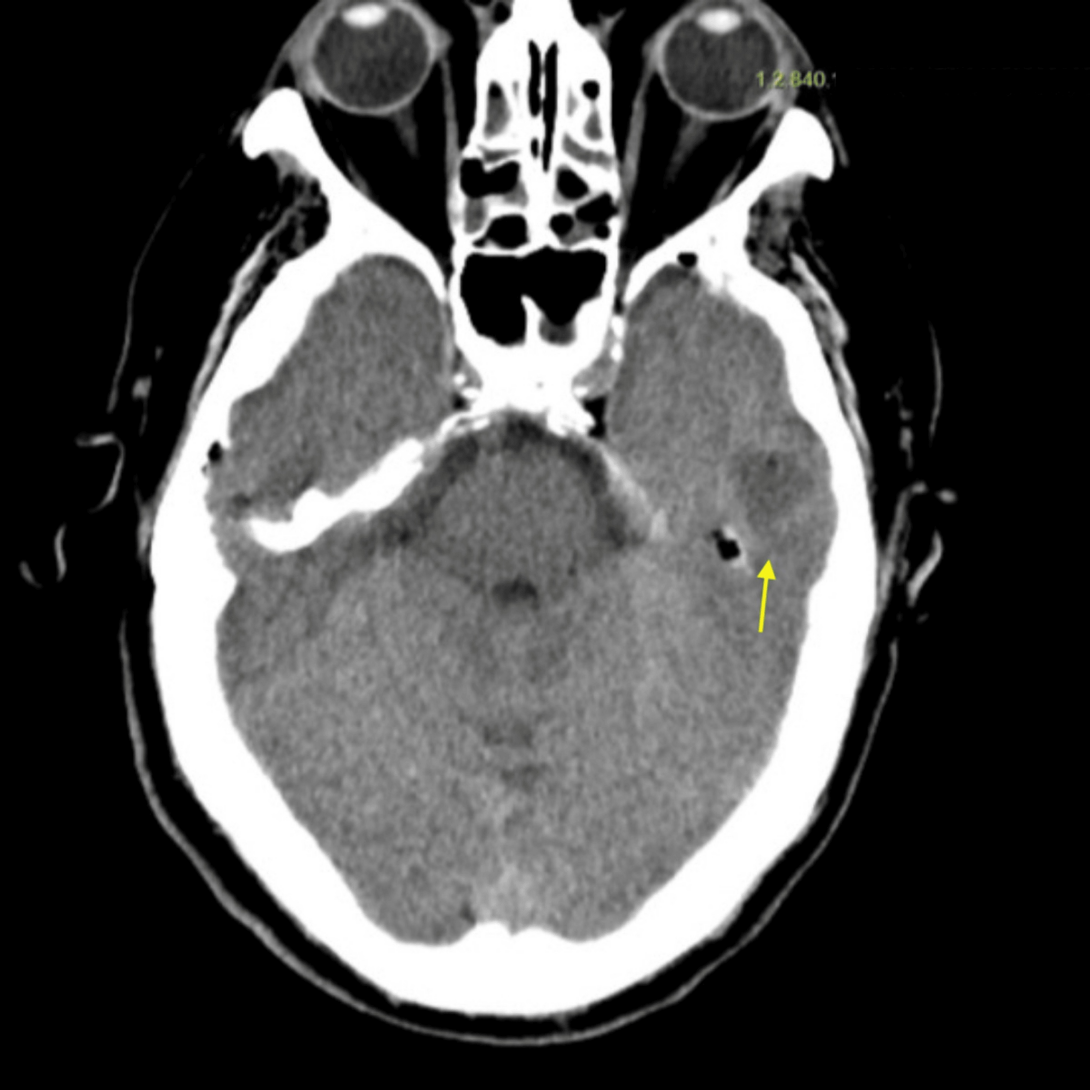
Cranial computed tomography (parenchymal window) Parenchymal-window images showing a hypodense area in the left temporobasal region adjacent to the petrous bone (yellow arrow), suggestive of early intracranial extension of otomastoiditis.

An adjacent hypodense area was observed in the left temporobasal cerebral parenchyma, suggestive of otomastoiditis complicated by early intracranial extension with an incipient temporobasal collection. No acute intracranial hemorrhage or significant mass effect was identified (Figure 2).

Brain magnetic resonance imaging (MRI) was not performed initially because of clinical instability, including hemodynamic compromise and depressed level of consciousness. Lumbar puncture was deferred due to hemodynamic instability, depressed consciousness, and concern for an intracranial space-occupying lesion on imaging.

Blood cultures obtained as part of the sepsis work-up grew Streptococcus pneumoniae, and urinary antigen testing for S. pneumoniae was also positive. The isolate was susceptible to ceftriaxone. Differential diagnoses considered at presentation included viral encephalitis, metabolic encephalopathy, and acute cerebrovascular disease.

Empirical intravenous antimicrobial therapy was initiated with ceftriaxone, vancomycin, ampicillin, and acyclovir. Intravenous dexamethasone was administered (8 mg in the ED, followed by 4 mg every 12 hours for three days). Antimicrobial therapy was subsequently deescalated to ceftriaxone monotherapy according to susceptibility results and continued intravenously for a total of ten days.

The patient was admitted to the intensive care unit (ICU) on the same day due to depressed consciousness and systemic instability. During the ICU stay, he required close neurological monitoring with repeated electroencephalograms because of suspicion of non-convulsive status epilepticus, which was treated with antiepileptic medication. The otorhinolaryngology team performed surgical drainage of the left mastoiditis, and S. pneumoniae was isolated from intraoperative mastoid samples, confirming the otogenic source of infection.

**Figure 3:**
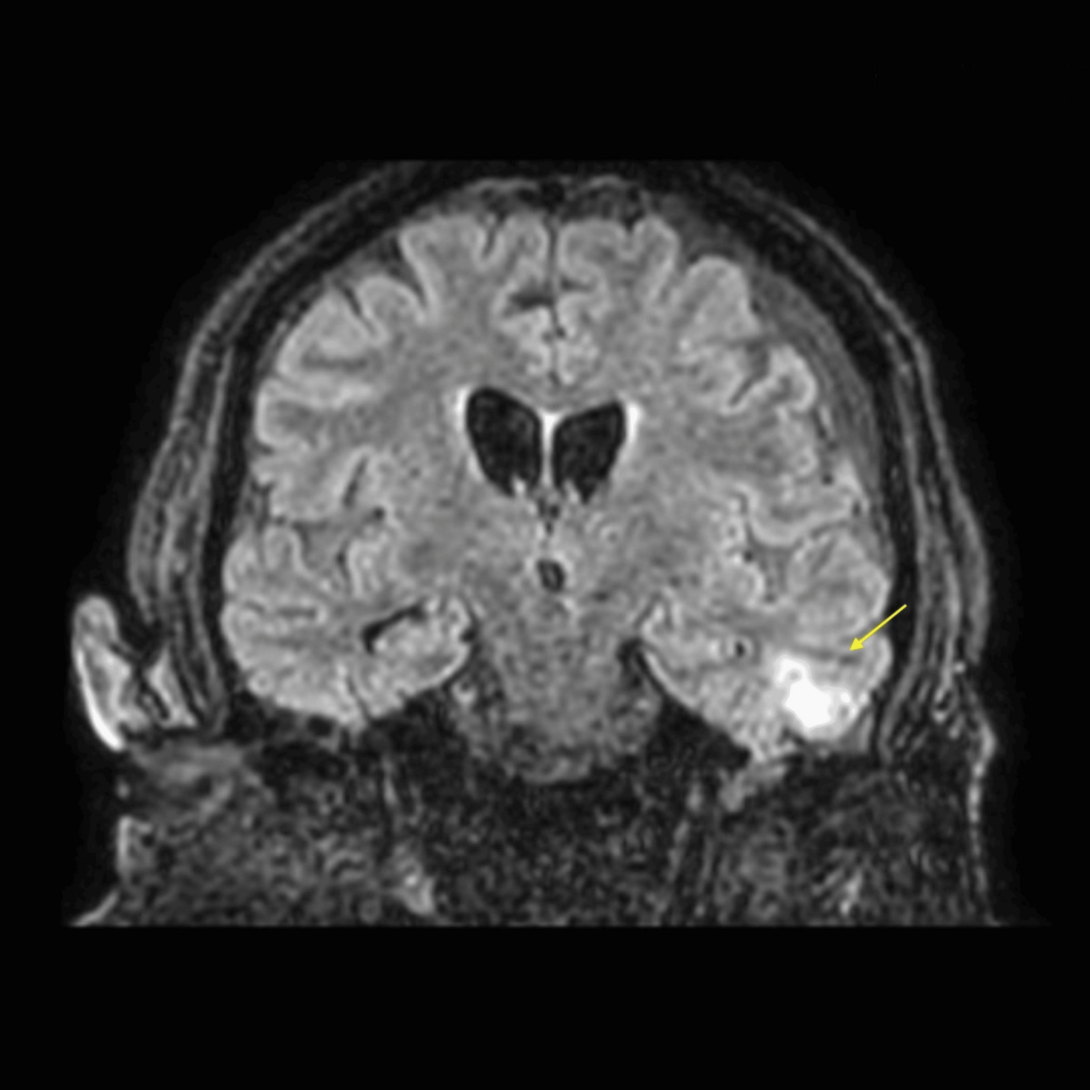
Brain magnetic resonance imaging Magnetic resonance imaging showing a left frontotemporal subdural collection and a left temporobasal abscess with mild surrounding edema (yellow arrow), associated with bilateral otomastoiditis and left-sided petrous bone erosion, consistent with otogenic intracranial spread.

Once clinically stabilised, brain MRI was performed. This demonstrated a left frontotemporal subdural collection and a left temporobasal abscess with mild surrounding edema. In addition, bilateral otomastoiditis was observed, with left-sided predominance and evidence of anterior petrous bone erosion. These findings were consistent with otogenic intracranial spread of infection (Figure 3).

The clinical course in the ICU was prolonged and complicated by acute kidney injury, nosocomial pneumonia, and the need for tracheostomy due to prolonged respiratory failure and the anticipated need for long-term airway support during neurological recovery. Laboratory investigations during hospitalization demonstrated persistent inflammatory activity and progressive metabolic abnormalities (Table 1).

Gradual neurological improvement was observed over the following weeks. The patient was transferred from the ICU to the neurology ward six weeks later. During the stay in the neurology ward, he showed slow but progressive neurological recovery. At discharge from acute care, he exhibited persistent cognitive deficits affecting memory, attention, language fluency, and executive functions, without significant focal motor deficits. Functional status at that time corresponded to a modified Rankin Scale score [8] of two indicating slight disability but functional independence. Given the persistence of neurological sequelae, the patient was transferred to a specialized rehabilitation hospital five weeks later. He had not received pneumococcal vaccination prior to this episode.

The clinical presentation, neuroimaging findings, and microbiological confirmation supported the diagnosis of pneumococcal meningoencephalitis secondary to acute mastoiditis.

## Discussion

This case illustrates the diagnostic challenges posed by otogenic intracranial infection in elderly patients presenting with non-specific symptoms and initially normal otoscopic findings. Adult otogenic meningitis may lack classical otological manifestations, leading to under-recognition of the infectious source and delayed diagnosis [2-4]. Such atypical presentations are particularly relevant in older adults, in whom neurological deterioration may be the predominant or initial manifestation of severe infection [3].

Masked mastoiditis, defined by the absence of overt otological signs despite active mastoid infection, represents an important diagnostic pitfall in the ED. Normal otoscopy may provide false reassurance and delay further evaluation. This atypical presentation has been increasingly recognized in adult and elderly patients with severe intracranial complications, including meningitis and meningoencephalitis [3,4].

Similar cases of masked mastoiditis presenting with intracranial complications have been described in the literature. For example, Omura et al. [3] reported meningoencephalitis caused by masked mastoiditis in an elderly patient with diabetes mellitus, highlighting the diagnostic challenge posed by normal otoscopic findings. Likewise, Liourdi et al. [4] described pneumococcal otogenic meningitis complicated by pneumocephalus and coma. These reports, together with the present case, emphasize the importance of considering an occult otogenic source in adults presenting with unexplained neurological deterioration.

Although acute mastoiditis is uncommon in adult emergency settings, it carries a high risk of serious complications [5]. Emergency medicine literature characterizes mastoiditis as a low-prevalence, high-risk condition requiring a high index of suspicion, particularly in patients presenting with fever and altered mental status without an obvious source of infection [5]. Compared with pediatric cases, adult otogenic infections are associated with a higher frequency of intracranial extension [2].

Neuroimaging played a pivotal role in establishing the diagnosis in this case. When otoscopic findings are normal or inconclusive, imaging becomes essential to identify an occult otogenic focus. High-resolution CT is useful for detecting mastoid opacification and cortical bone erosion, while MRI provides superior characterization of intracranial complications such as meningitis, subdural collections, and brain abscess [6,7]. In critically ill patients, imaging may need to precede lumbar puncture to avoid diagnostic delay and potential complications [4,5].

In severe otogenic infections, early empirical antimicrobial therapy combined with imaging-based diagnosis may provide sufficient diagnostic certainty, particularly when microbiological confirmation is obtained from blood cultures or surgical specimens [4].

S. pneumoniae remains the most frequently identified pathogen in adult otogenic meningitis. Contemporary series report pneumococcus in approximately 70-75% of cases, despite the availability of pneumococcal conjugate vaccines [2]. These findings highlight the continued clinical relevance of pneumococcal otogenic infections in adults and the persistent vulnerability of elderly and high-risk populations. In this case, the absence of prior pneumococcal vaccination may have contributed to the disease severity.

The patient experienced a prolonged and complicated clinical course, including persistent cognitive impairment. Advanced age, altered consciousness at presentation, comorbidities such as diabetes mellitus, and intracranial extension have been associated with poorer outcomes in otogenic intracranial infections [9]. These factors were all present in our patient and may explain the incomplete neurological recovery observed.

Surgical drainage of the mastoid focus represented a key component of management in this case. Although most surgical recommendations derive from paediatric series, current evidence supports surgical intervention in complicated mastoiditis with intracranial extension to achieve source control and prevent further neurological deterioration [5,10]. This case underscores the importance of early multidisciplinary collaboration among emergency physicians, otolaryngologists, intensivists, and infectious disease specialists [9].

Overall, this case reinforces a key clinical message: normal otoscopic findings do not exclude significant otogenic disease. In elderly patients presenting with fever and altered mental status, clinicians should consider masked mastoiditis as a potential source of invasive intracranial infection and pursue timely neuroimaging when clinical suspicion persists.

## Conclusions

Masked mastoiditis represents an important diagnostic pitfall in elderly patients presenting with fever and altered mental status, particularly when otological symptoms and otoscopic findings are absent. Delayed recognition of the otogenic focus may lead to severe intracranial complications such as pneumococcal meningoencephalitis. Normal otoscopy does not exclude significant otogenic disease. Early consideration of an occult otogenic focus and timely neuroimaging are essential, particularly in clinically unstable patients in whom lumbar puncture may need to be deferred. Increased awareness of this diagnostic pitfall may help reduce morbidity associated with invasive pneumococcal infections in high-risk adults.
